# Asthma Symptoms in Bakeries at Parakou, Benin

**DOI:** 10.1155/2020/3767382

**Published:** 2020-01-30

**Authors:** Serge Ade, Mênonli Adjobimey, Gildas Agodokpessi, Marie Sylvie Kouassi, Fabien A. Gounongbe, Ibrahim Cisse, Spero H. R. Hounkpatin

**Affiliations:** ^1^Faculty of Medicine, University of Parakou, Parakou, Benin; ^2^International Union Against Tuberculosis and Lung Diseases, Paris, France; ^3^Faculty of Health Sciences, University of Abomey-Calavi, Cotonou, Benin; ^4^National and Teaching Hospital of Respiratory Diseases, Cotonou, Benin

## Abstract

*Background and Objectives*. There is a dearth of information on asthma among bakers in low-income settings. The objectives of this study were to determine (i) the prevalence of asthma symptoms, (ii) factors associated with probable occupational asthma (OA), and (iii) work habits that might lead to a dusty workplace environment, Parakou, Benin. *Materials and Methods*. This was a mixed methods (cross-sectional quantitative and qualitative) study carried out between March and September 2018.

**Results:**

Of 210 employees/apprentices in 26 bakeries, 190 (91.48%) were included in the study: median age was 25.50 (IQR = 22 − 32) years, 157 (82.63%) were aged <40 years, and the male-to-female ratio was 26.14. Of these, 111 (58.42%) worked in a salted bread and 79 (41.58%) in a sweet bread bakery. An asthma history was reported by 3.68%. Symptoms consistent with asthma, work-related asthma, OA, and work-aggravated asthma were found in 13.68%, 12.63%, 10%, and 2.63%, respectively. Asthma confirmation was obtained in 15.79% of bakers with probable OA and in 23.08% of all bakers with suspected asthma. A history of allergic rhinoconjunctivitis was associated with probable OA (aOR = 106; 95%CI = 17.79 − 2093; *p* < 0.001). Of the 24 bakers with probable work-related asthma, 3 (12.50%) were prescribed a short-acting beta2-agonist and 2 (8.33%) an inhaled corticosteroid. No worker had had a systematically planned annual medical visit; some habits at work were identified as leading to flour and dust suspension at the workplace.

**Conclusion:**

Clinical manifestations of OA were common among bakers in Parakou and were associated with allergic rhinoconjunctivitis. There is a need to improve technical preventive measures and treatment, as well as to institute systematic medical visits for these workers.

## 1. Introduction

The baker's profession is well known to be strongly associated with occupational asthma (OA) worldwide, including in low- and middle-income countries (LMICs) [1–3]. Numerous allergens in a bakery may induce sensitization and allergic reactions. Particles that are derived from wheat, rye, and oats are reported to be highly allergenic. Wheat, the most common flour used for bread making, contains more than forty allergens, with water-soluble globulins and albumins thought to be the most allergenic. Baker's yeast, fungal alpha-amylase, improvers such as lecithin, soy flour, additives such as malted products, and contaminants that include storage mites and moulds are also sources of sensitization in bakeries [1, 4, 5].

The prevalence of asthma symptoms appears to be high among bakers. In a study carried out in France on artisanal bakery employees, the prevalence of asthma symptoms was nearly 17% [6]. Work-related asthma (WRA) is estimated to affect 5 to 10% of bakers [4], and this includes OA and worked-aggravated asthma (WAA). OA occurs in workers with no previous asthma whose first symptoms are initiated by exposure to agents at work. WAA is characterised by a worsening of symptoms of asthma in a worker with previously known asthma before any exposure to the work-related agents [7]. In a study conducted in Cape Town, the prevalence of OA was 13% and that of WAA was 3% [8].

In LMICs, similar to other OAs, baker's asthma is either underdiagnsoed or diagnosed late [3], due to several reasons that include lack of knowledge about disease symptoms and fear of job loss [1, 3]. This has harmful consequences not only for the individual but also for the whole family and systematic medical visits for workers are often missed [3]. Furthermore, recommended diagnostic tools such as specific bronchial challenges or immunological testing to specific allergens present in the workplace [7] are often not available. With challenges in implementing medical and technical preventive measures in these countries, we hypothesize that the burden of OA and WAA may be frequent among bakers despite the low number of studies in the literature from these settings, especially from sub-Saharan Africa.

The objectives of this preliminary study were to determine the prevalence of asthma manifestations, factors associasted with OA, and work habits that promote increased dust in the workplace among bakers in Parakou city in Benin.

## 2. Materials and Methods

### 2.1. Study Design

This was a mixed cross-sectional quantitative and qualitative study using data that were prospectively collected between March and September 2018.

### 2.2. Setting

The study was carried out in salted and sweet bread bakeries in Parakou, a city located in the north-central part of Benin with a population of 255,478 in 2013 [9]. Theoretically, Parakou has 37 bakeries, 17 produce salted and 20 produce sweet bread. However, only 11 (64.71%) salted and 15 (75%) sweet bread bakeries were really functional and these were all visited. In Parakou, similar to the rest of the country, baker's asthma is recognised as an occupational disease by Table no. 72 of the list of occupational diseases (unpublished data).

### 2.3. Study Population

All employees and apprentice bakers who gave their formal consent were included in the study. For those under 18 years of age, informed consent from a parent (or guardian) was obtained prior to the survey.

### 2.4. Data Collection and Principal Variables

For each individual worker included in the study, information was anonymously collected on demographic characteristics, comorbidities, type of bakery worked in, duration in the profession, position in the company, and presence of asthma symptoms. Bakers were then examined; and in those who reported asthma symptoms, their onset or aggravation during working hours at the bakery and their improvement during holidays were investigated. Bakers were then examined using spirometry with Spirodoc® (serial number: SN A23-0W.00398), followed by reversibility testing after inhalation through a chamber of salbutamol 400 *μ*g dosage. The tests were performed by a well-trained medical student according to American Thoracic Society (ATS) and European Respiratory Society (ERS) guidelines [10]. Results were interpreted by an experienced pulmonologist and in line with ATS and ERS guidelines [11].

In addition, workers' habits in their respective positions in the bakery were observed during manufacturing of the bread in order to identify any practices that might promote flour dusting in the bakery. All this information was recorded in a data documentation form filled out by the investigator.

### 2.5. Diagnostic Criteria

1. *Asthma Symptoms*. Asthma symptoms considered in this study included the four cardinal symptoms of the disease, namely, dyspnoea, wheezing, coughing, and chest tightness [12]
2. *Reversible Obstructive Pulmonary Disorder*. This was defined by comparing the individual ratio of forced expiratory volume in one second (FEV1) and vital capacity (VC) to the lower limit of normal; and significant reversibility was diagnosed by an increase in FEV1 ≥ 12% and 200 ml in absolute values. The extrapolations that have been proposed to derive African standards were used to determine theoretical values [10, 11]
3. *Confirmed Asthma*. The diagnosis was made in a symptomatic worker with a reversible obstructive disorder [12]
4. *History of Asthma*. Any worker who reported having asthma symptoms in the past or was diagnosed with asthma by a medical doctor or was taking asthma medication before starting work at the bakery
5. *Probable OA*. A worker who reported (a) never having had asthma symptoms before starting baking; (b) triggering of symptoms during working hours; and (c) improvement in symptoms while on holiday [7]
6. *Probable WAA*. A worker who reported (i) a history of asthma before starting work at bakery; (ii) worsening of symptoms during work hours at bakery; and (iii) improvement while on holiday [7]
7. *Probable Work-Related Asthma (WRA)*. Association of OA and WAA manifestations [7]
8. *Probable Asthma in Bakery*. Association of WRA and nonwork-related asthma
9. *Allergic Rhinitis*. The diagnosis was based on symptoms and referred to repeated symptoms of sneezing, rhinorrhea, nasal pruritus, and a blocked nose triggered by some factors and without evidence for any respiratory infection [13]

### 2.6. Statistical Analysis

The data were entered into the EpiData Entry client software v2.0.7.7.22 (EpiData Association, Odense, Denmark). Data were analyzed using software R version 3.6.0. Percentages were calculated to describe qualitative variables, medians (and interquartile range (IQR)) for quantitative variables. Factors associated with probable OA were investigated by univariate analysis and then multivariate analysis using multiple logistic regression. All exposure factors with a *p* value ≤ 0.10 on univariate analysis were included in the multivariate analysis. The crude and adjusted odds ratios, their 95% confidence interval (95% CI), and the *p* values were determined. Levels of significance were set at 5%.

### 2.7. Ethical Considerations

The study was approved by the Local Ethics Committee for Biomedical Research of the University of Parakou, Benin.

## 3. Results

### 3.1. Characteristics of Bakers and Prevalence of Asthma Symptoms and of Confirmed Asthma

Overall, of the 210 employees or apprentices that were identified in the 26 bakeries, 190 (91.48%) consented to participate in the survey. The median age was 25.50 (IQR = 22 − 32) years, 157 (82.63%) were aged less than 40 years, and the male-to-female ratio was 26.14. Of these bakers, 111 (58.42%) worked in a salted bread and 79 (41.58%) in a sweet bread bakery, with a median duration of working in the bakery being 5 (IQR = 1 − 11.5) years. There were 94 (49.47%) who were employed in priority as processors/laminators (Table 1).

The prevalence of a history of asthma was 3.68%. The prevalence of probable WRA was 12.63%. More specifically, the prevalence of probable OA and WAA was 10% and 2.63%, respectively. Of the 24 bakers with probable WRA, 3 (12.50%) were prescribed a short-acting beta_2_-agonist and 2 (8.33%) an inhaled corticosteroid. The prevalence of probable asthma in bakeries was 13.68% (Tables 2 and 3). The prevalence of confirmed asthma was 15.79% among workers with probable OA and 23.08% among all bakers with probable WRA (Table 4).

### 3.2. Characteristics and Factors Associated with Probable OA

The median age of workers with probable OA was 24 (IQR = 20 − 30) years. They were all males. In 5 (26.32%) workers, respiratory symptoms were preceded by nasal and ocular symptoms. Three workers (15.79%) had bilateral wheezing during pulmonary auscultation at the time of the survey. Ten (52.63%) workers were shapers/rolling mill operators, 6 (31.58%) were oven bakers, and 3 (15.79%) were kneaders. The median duration of working in a bakery was 4 (IQR = 1 − 13) years and range 48 days to 20 years. At univariate analysis, an association between OA and factors such as age, sex, history of family asthma, allergic rhinoconjonctivis, eczema, smoking, type of bakery, and duration of bakery working was sought. The position was not included among the potential risk factors since this was not specific to a designated worker. They usually have a frequent turnover between the different positions. After multiple logistic regression, a history of allergic rhinoconjunctivitis was associated with probable OA (aOR = 1.06.10^2^; 95%CI = 17.79 − 2093; *p* < 0.001), after adjustment for eczema and type of bakery (Table 5).

### 3.3. Annual Medical Check-Up and Work Habits Assessment

None of the workers who were interviewed had, to date, systematically planned any annual medical visits for the checking of asthma symptoms combined with respiratory measurements such as peak flow or spirometry. No personal protective equipment was used by workers and no specific ventilation system was in place.

Additionally, there were some habits and behaviours favouring the suspension of flour and dust in the air, mainly occuring during the mixing process. Of the 26 bakeries that were visited, the emptying of the flour bag was not done gently in two (8%). In 12 bakeries, water was not poured into the mixer before the flour. In salted bread bakeries, the flour was spilled into the mixer before the ice cubes. At the end of the emptying of the bag into the mixer, the officer tapped it to make sure that all the flour was poured. The flour that was not yet completely immersed in the water lifted up as soon as the mixer was started. The baker became sprinkled with flour several times: during the emptying of the mixer tank, on the dough pieces before the division, the planks, the work surface during shaping, the shovel, and the breads before baking. Sweeping and cleaning were not done in a wet environment, resulting thereby once again in flour and dust suspension.

## 4. Discussion

The objectives of this study were to determine the prevalence of clinical asthma manifestations, factors associated with symptoms suggestive of OA and work habits and behaviours that might promote increased dust in the workplace among bakers in Parakou city, a sub-Saharan African setting. One of the strengths of the study was the recruitment of almost all the bakers who worked in this town, with the inclusion of more than nine tenths of them in the study. In addition, data on the different variables were prospectively collected, reducing thereby missing information-related bias. The main limitation of the study was the inability to confirm the diagnosis of baker's asthma. As mentioned above, specific bronchial challenge testing and investigation of the sensitization to allergens present in the occupational environment could not be carried out in our routine working conditions, due to the weakness of our technical platforms. We tried to perform serial measurements of peak expiratory flow during both working days and holidays to confirm the diagnosis of OA [7]. Unfortunately, this was found time-consuming by the bakers, who were mostly daily workers and were paid proportionally to their achievement at work. Moreover, the study did not receive any funding to motivate them by providing some incentives. Despite these limitations, the study showed some interesting findings.

The high male predominance among the workers who were investigated has been reported in other studies [14]. Workers were also relatively young, with a median age of 25.50 years and over 80% aged less than 40 years. Older workers may have stopped working earlier, due to the occurrence of occupational diseases including OA. It was not possible, in this study, to investigate the real reasons why each former worker stopped working at bakery.

The prevalence of asthma symptoms among bakers in Parakou was 13.68%, slightly lower than that found by Gillet in 2015 in France, 17% [6]. However, the proportion found was much higher than that reported in the general population in this city, 3.6% [15]. The prevalence was comparable to that reported by Sigari et al. from Sanandaj, Iran, in 2006, at 13% [14]. The prevalence of probable OA and WAA was 10% and 2.63%, respectively. The same trend was reported by Baatjies et al. in a study carried out in Cape Town between 2003 and 2004. These authors found 13% and 3%, respectively, for OA and WAA. In their study, in addition to clinical manifestations, the diagnosis was made using nonspecific bronchial hyperreactivity and skin testing for wheat, rye, barley, maize, *Lepidoglyphus destructor* (a storage mite), fungal amylase, and/or specific IgE isolation [8].

Respiratory symptoms among bakers are frequent according to many studies conducted in developed countries [16]. Bakers and confectioners are those most commonly affected by OA in France, with an annual incidence of 683 cases per million workers [17]. In Norway and the United Kingdom, exposure to grain and flour dust is reported to be the second leading cause of OA [4, 18, 19]. Over the period 1992-1997, the incidence of asthma among bakers in the United Kingdom was estimated at 951 cases per million people per year [19]. In Norway, the incidence of baker's asthma was 2.4 and 1 case per 1000 person-years, respectively, for men and women [18]. Worse, the number of asthma cases among bakers was thought to have increased in recent years [4], probably due to a better appropriation of symptoms and diagnostic tools.

In our setting, the true proportion of bakers with WRA or OA may be even higher than that reported in this study, since some employees, fearing to lose their job, may not have declared their symptoms, contributing to an underestimation of the disease. Additionally, some bakers are not administratively declared and therefore cannot be correctly followed-up. Another possible cause that may contribute to underestimating this prevalence is lack of recognition of asthma symptoms by some bakers.

Considering the challenges in achieving serial measurements of peak expiratory flow, a flow-volume curve with reversibility was performed in all symptomatic workers or those with a previous history of asthma. The diagnosis of asthma was confirmed among 15.79% of workers suspected of having OA and among 23.08% of all bakers with probable asthma. The absence of obstructive disorder between attacks was not surprising and has also been previously reported by others [12, 20].

After logistic regression, rhinoconjunctivitis was associated with OA after having adjusted for other factors. Several authors had highlighted the role of atopy in promoting the occurrence of OA [1]. Baker's asthma is preceded by allergic rhinitis, as it is often the case with sensitization to high molecular weight agents [1, 8, 16]. Adequate treatment of this comorbidity is strongly recommended; otherwise, it may worsen asthma symptoms and prevent the control of the disease [7, 16]. New workers who already have allergic diseases should be better informed on the high risk of developing asthma in bakeries, especially if preventive measures are not implemented. Such patients also require special attention during follow-up visits.

Finally, assessment of workers' habits revealed many behaviours that promote increased dust at the workplace during the many stages of bread making. There is need for urgent implementation of relevant technical prevention measures in these bakeries. Taking into account local resources, this may include delicately placing flour bags, not tapping the bag, and using a mixer at a slow speed until the flour is mixed into the liquid ingredients. Cleaning of floors and work surfaces after their humidification should also be advocated. Additionally, although this was not investigated in our study, we recommend the introduction of an effective ventilation system to reduce the air concentration of particles. Storing flour in the right conditions of temperature and humidity that inhibit the growth of microorganisms can be recommended. Other actions would include use of personal protective equipment such as masks. Flour exposure limit values are proposed in some countries [21, 22]. Training of exposed workers is essential for behavioural change, and according to some authors, this could effectively reduce dust in the workplace by 54% [9]. Finally, medical follow-up visits with regular measurement of respiratory volume and air flow must be instituted in every bakery, a unique way to ensure early, total, and definitive eviction. In the same way, it is urgent to improve treatment among symptomatic patients, since the proportion of those undergoing treatment was low before the survey.

## 5. Conclusion

Clinical manifestations of OA were common among bakers in Parakou, and they were associated with allergic rhinoconjunctivitis. Many of the behaviours during bread making promoted increased dust by flour, and these should be corrected by strengthening technical prevention measures. Finally, we advocate for systematic annual follow-up visits of workers in this sector.

## Figures and Tables

**Table 1:** Characteristics of workers investigated in bakeries at Parakou, Benin, March–September 2018.

	Number	(%)
Gender		
Males	183	(96.32)
Females	7	(3.68)
Age		
Median age (year)	25.50	
Interquartile range (year)	22-32	
Range (year)	13-66	
Tobacco status		
Nonsmoker	181	(95.26)
Current smoker	8	(4.21)
Ex-smoker	1	(0.53)
Allergic rhinitis	36	(18.95)
Allergic conjunctivitis	28	(14.74)
Eczema	11	(5.79)
Family atopy	10	(5.26)
Type of bakery		
Salted bread bakery	111	(58.42)
Sweet bread bakery	79	(41.58)
Duration of bakery working		
Median (year)	5	
Interquartile range (year)	1–11.5	
Range (year)	0.1-44	
Position at the time of the survey in the bakery		
Shaper/laminator	94	(49.47)
Kneader	51	(26.84)
Oven baker	29	(15.26)
Cashier	13	(6.84)
Cleaner	3	(1.58)
Total	190	

**Table 2:** Prevalence of asthma symptoms and evolution during working hours of bakers at Parakou, Benin, March–September 2018.

	Number	(%)
Asthma symptoms		
Dyspnea		
Absent	166	(87.37)
Only during working hours	19	(10)
Already present but worsened during working hours	5	(2.63)
Wheezing		
Absent	186	(97.89)
Only during working hours	2	(1.05)
Already present but worsened during working hours	2	(1.05)
Cough		
Absent	176	(92.63)
Only during working hours	9	(4.74)
Already present but worsened during working hours	5	(2.63)
Chest tightness		
Absent	180	(94.74)
Only during working hours	7	(3.68)
Already present but worsened during working hours	3	(1.58)

**Table 3:** Prevalence of asthma symptoms, of work-related and nonwork-related asthma symptoms in bakeries at Parakou, Benin, March–September 2018.

	Bakers with asthma symptoms	%
History of asthma	7	(3.68)
Bakers with asthma symptoms or history of asthma	26	(13.68)
Probable work-related asthma	24	(12.63)
Probable occupational asthma	19	(10)
Probable work-aggravated asthma	5	(2.63)
Probable non work-related asthma	2	(1.05)
Total	190	

**Table 4:** Spirometry results of bakers with asthma symptoms or an history of asthma at Parakou, Benin, March–September 2018.

	Total	Spirometry done	OVD^∗^ reversible (%^$^)
Bakers with asthma symptoms or history of asthma	26	26	6 (23.08)
Probable work-related asthma	24	24	5 (20.83)
Probable occupational asthma	19	19	3 (15.79)
Probable work-aggravated asthma	5	5	2 (40)
Probable nonwork-related asthma	2	2	1 (50)

^∗^ OVD = obstructive ventilatory disorder. ^$^Denominator = number of spirometry performed.

**Table 5:** Factors associated with probable occupational asthma, after univariate and multiple logistic regression analysis among bakers at Parakou, Benin, March–September 2018.

	*n*/*N*	%	Bivariate analysis		Multivariate analysis		
			cOR	*P* value	aOR	IC 95% aOR	*P* value
Age			0.98	0.567			
Sex				0.992			
Female	0/7	(0)	1				
Male	19/179	(10.61)	5.10.10^6^				
Family asthma				0.982			
No	18/176	(10.23)	1				
Yes	1/10	(10)	0.98				
Allergic rhinoconjunctivitis				<0.001			
No	1/144	(0.69)	1				
Yes	18/42	(42.86)	1.07.10^2^		106	17.79 - 2093	<0.001
Eczema				<0.001			
No	13/175	(7.43)	1				
Yes	6/11	(54.55)	14.95		2.14	0.51 - 9.50	0.300
Smoking				0.240			
No	17/177	(9.60)	1				
Yes	2/9	(22.22)	2.69				
Type of bakery				0.067			
Sweet bread bakery	4/77	(5.19)	1		1		
Salted bread bakery	15/109	(13.76)	2.91		0.60	0.12 - 2.95	0.519
Duration of bakery working			0.98	0.534			

cOR = crude odds ratio; aOR = adjusted odds ratio; 95% CI = 95% confidence interval.

## Data Availability

The quantitative data used to support the findings of this study are available from the corresponding author upon request.
